# Approach to the Diagnosis and Management of Infected Pancreatic Necrosis: A Narrative Review

**DOI:** 10.7759/cureus.83020

**Published:** 2025-04-25

**Authors:** Saroj K Sahu, Suprabhat Giri, Swati Das, Chinmaya Deepak Patro, Dibya L Praharaj, Bipadabhanjan Mallick, Preetam Nath, Sarat Chandra Panigrahi, Anil C Anand

**Affiliations:** 1 Gastroenterology and Hepatology, Kalinga Institute of Medical Sciences, Bhubaneswar, IND; 2 Radiodiagnosis, Kalinga Institute of Medical Sciences, Bhubaneswar, IND

**Keywords:** acute necrotizing pancreatitis, endoscopic ultrasound-guided biliary drainage, infected pancreatic necrosis, percutaneous catheter drainage, step-up approach

## Abstract

Infected pancreatic necrosis (IPN) is a dreaded complication of acute necrotizing pancreatitis and is linked to persistent organ failure, sepsis, and increased morbidity and mortality. Clinical indicators of IPN include fever, clinical deterioration, and worsening inflammatory markers. The diagnosis of IPN is based on clinical signs, microbiological confirmation, and radiological evidence, with contrast-enhanced CT being the preferred imaging modality. In the absence of tests with high sensitivity, a high clinical suspicion is required for early recognition and treatment. Although there is no way to prevent IPN, a systematic management approach with parenteral antibiotics, nutritional management, and minimally invasive procedures has become the cornerstone of the treatment. The step-up approach includes minimally invasive procedures that minimize procedure-related complications and are associated with improved outcomes. The percutaneous route remains the most common route for drainage, while endoscopic interventions are preferred for perigastric or periduodenal encapsulated collections. The use of lumen-apposing metal stents is associated with excellent outcomes in cases of infected walled-off necrosis. Patients with significant quantities of infected necrosis may benefit from direct or percutaneous endoscopic necrosectomy. Minimally invasive surgical techniques followed by open surgeries are reserved for patients who do not improve with percutaneous or endoscopic necrosectomies. The outcome can be maximized through a multidisciplinary approach by a team of interventional radiologists, advanced therapeutic endoscopists, and surgeons.

## Introduction and background

Acute necrotizing pancreatitis (ANP) is a severe form of acute pancreatitis (AP) and accounts for 15-20% of cases. The mortality rate in the ANP is quite high (20-30%) [1]. Around one-third of cases of ANP develop infected pancreatic necrosis (IPN), which is associated with persistent organ failure (OF), sepsis, high morbidity, and mortality [2]. The mortality rate of IPN with OF is twice as high as that of IPN without OF [3,4]. Patients with > 50% pancreatic necrosis exhibited the highest rates of OF, IPN, and mortality. Apart from bacterial causes, fungal infection can also be associated with pancreatic necrosis, the prevalence of which is influenced by factors such as the severity of pancreatitis, the duration of hospitalization and intensive care unit (ICU) stay, use of broad-spectrum antibiotics, and invasive procedures [5]. A study reported a high proportion of fungal pancreatic infections affecting 46% of patients with IPN or pseudocysts, with 24% of patients having fungal infections at the first microbiological sampling (primary infection), and an additional 22% developing them later (secondary infection) [5]. A meta-analysis of 22 studies involving 2,151 patients with necrotizing pancreatitis found a mean incidence of fungal infection of 26.6%, associated with a prolonged duration of hospital and ICU stay with increased mortality [6]. Henceforth, IPN almost always requires an invasive intervention. Current guidelines recommend a "step-up" approach for IPN [7]. The present review aims to summarize the current evidence on the diagnosis and management of IPN.

## Review

Diagnostic evaluation of IPN

IPN was considered typically a late event during AP; however, one-fourth of patients with ANP also develop IPN in the early phase (7-14 days, early IPN) [8]. Clinical indicators of IPN are fever, persistent unwellness, clinical deterioration, bacteremia, or worsening laboratory markers of inflammation. Persistent SIRS for a longer duration in the early phase of AP is associated with a higher incidence of IPN [9]. In the late phase of AP, clinical signs of infection, i.e., persistent systemic inflammatory response syndrome (SIRS) or OF, late-onset (secondary) OF, or clinical deterioration after an initial improvement, are considered signs of IPN [10,11].

Microbiological Evaluation

Microbiological confirmation is shown either by a positive Gram stain or culture from the aspirated/drained necrotic tissue. Fine needle aspiration (FNA) cultures guided by ultrasound (USG), computed tomography (CT), or endoscopic ultrasound (EUS) are highly specific for IPN. However, false-negative results can occur in 20%-25% of cases, making the diagnosis challenging. Ideally, FNA should be performed prior to starting broad-spectrum antibiotics to allow for targeted therapy based on culture results. Repeat FNA may be considered if clinical suspicion remains high despite an initial negative result. The risk of introducing infection into a sterile collection through FNA is considered very low (<1%) [12]. However, current guidelines do not recommend FNA for Gram-stain and cultures in all cases [13,14].

Radiological Evaluation

**Figure 1:**
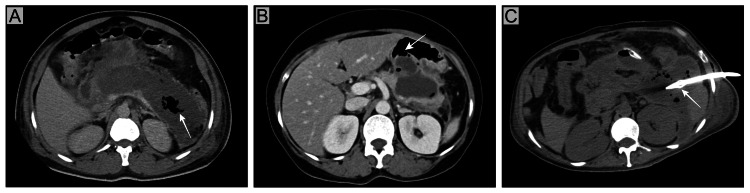
Air inside the pancreatic necrosis is most commonly due to (A) infection (as evident by gas inside the collection - arrow) but can also be due to (B) fistulizing communication with the gastrointestinal tract (arrow) and (C) intervention (arrow showing percutaneous catheter). Image credits: Dr. Suprabhat Giri, Dr. Swati Das, and Dr. Chinmaya Deepak Patro Patients' consent was taken for the publication of the image.

Contrast-enhanced CT (CECT) is the preferred imaging modality for diagnosing IPN and identifying signs of infection [14,15]. Radiologically, IPN is evident by the presence of a "bubble sign" (extra-intestinal air in the pancreatic or peripancreatic tissue) on a CT abdomen (Figure 1). The sensitivity of the "bubble sign" is low (56%), and the specificity is high (97%). It is also important to note that gas can be present due to the spontaneous rupture of the necrotic collection into the gastrointestinal (GI) lumen, leading to fistulization or prior interventions (Figure 1) [16]. If imaging does not show a bubble sign, the clinical features of infection guide the treatment. Magnetic resonance imaging (MRI) can provide a better distinction between solid necrotic and predominantly fluid collections. While frequently discussed for assessing fluid collections, its additional value for diagnosing IPN compared to CECT is less clear.

Serological Evaluation

**Figure 2:**
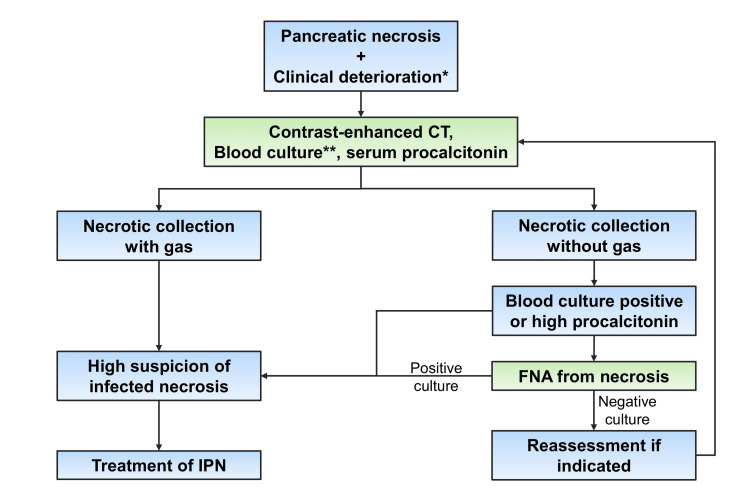
Step-wise approach to the diagnosis of infected pancreatic necrosis. *Clinical deterioration included the development of fever, worsening of systemic inflammatory response syndrome, or new onset organ failure. **Around 30% of patients with infected pancreatic necrosis can have a positive blood culture. Image credits: Dr. Suprabhat Giri

Corroborative evidence of IPN in the form of increased serum biomarkers of infection, such as procalcitonin (PCT), C-reactive protein (CRP), and total leucocyte count (TLC), aids in decision-making in these cases. Several biological markers, such as blood urea nitrogen (BUN), CRP, and PCT, have been studied to predict infected (peri)pancreatic necrosis. Mofidi et al., in a meta-analysis, found that, when the cut-off value of the serum PCT was greater than 0.5 ng/mL, the sensitivity and specificity of PCT for predicting IPN were 0.80 and 0.91, respectively [17]. Another study has also shown that a baseline (within 72 hours of hospitalization) > 0.5 ng/mL, especially PCT > 0.68 mg/dL, was predictive of infectious complications of ANP such as IPN, acute cholangitis, and extrapancreatic infections [18]. According to a prospective study by Samanta et al., patients with IPN and a baseline PCT ≥ 1.0 ng/mL had a substantially higher post-intervention mortality (26% versus 7%, p = 0.044) than those with a baseline PCT < 1.0 ng/mL [19]. A recently published meta-analysis observed that persistently elevated serum CRP and PCT were good predictors of infection in ANP with a pooled AUC of 0.88 and 0.86 for CRP and PCT, respectively [20]. These biomarkers are useful as a guide to antibiotics and the clinical success of therapeutic intervention in IPN [21]. Figure 2 outlines the approach to the diagnosis of an IPN.

Initial treatment of IPN

Role of Antibiotics

IPN develops several days after hospitalization. Gut-derived bacteria (small intestine) appear to be the primary source of enteral bacteria in IPN [22]. Bacterial translocation from the small intestine to infected necrosis occurs by the direct (bacterial translocation) or indirect (hematogenous) pathways. Multidrug-resistant organisms (MDROs) such as carbapenem-resistant *Klebsiella pneumoniae*, carbapenem-resistant *Acinetobacter baumanii*, and extended-spectrum beta-lactamase-producing *Escherichia coli* are associated with an increased risk of complications and mortality [23]. Although Gram-negative enteric bacteria are the predominant etiological agents of IPN, Gram-positive organisms such as *Enterococcus faecalis*, *Enterococcus faecium*, coagulase-negative *Staphylococcus*, and anaerobes can also cause IPN [24]. Extra pancreatic infections, such as bacteremia, respiratory tract infection, and urinary tract infection, also contribute to higher morbidity and mortality [25-27].

Prophylactic antibiotic therapy has no role in sterile necrosis. Broad-spectrum intravenous antibiotics are recommended when IPN is suspected or confirmed, with the choice of antibiotics depending on pancreatic tissue penetrability and the patient's condition. Antibiotics with good pancreatic penetration include metronidazole, fluoroquinolones, piperacillin-tazobactam, cefepime, and carbapenems. No single (or combination) antibiotic has a superior efficacy or safety profile than the other(s). Quinones have good pancreatic tissue penetrability, but they are often used as second-line agents due to widespread antibiotic resistance or in those with allergies to beta-lactam agents. One of the carbapenems, alone or in combination with metronidazole, is often preferred as a starting antibiotic regimen. However, due to the emergence of carbapenem-resistant organisms, the use of carbapenems should be optimized and should be used only in critically ill patients [13,14,28]. Antibiotics are typically administered for at least two weeks after they are started. The decision to stop antibiotics is usually taken after several (five to seven) days, once the cultures turn negative, along with the absence of clinical signs of infection. In the ICU setting, principles of antimicrobial stewardship apply, including paying attention to altered pharmacokinetics in critically ill patients, de-escalation of broad-spectrum therapy once cultures are available, and early withdrawal of antibiotics once source control has been established [29].

Routine antifungal therapy is not indicated in IPN. *Candida albicans* is the most frequently encountered organism in pancreatic necrosis, responsible for approximately two-thirds of the cases [6]. Thus, for patients with fungemia or culture-positive fungal infections, echinocandins are recommended over azoles, even if they are azole-sensitive [30]. Patients with ongoing sepsis and organ failure, despite optimum antibiotic therapy, need definitive treatment with drainage and debridement.

Nutritional Management

In patients with AP and ANP, early (usually within 24 hours after admission) enteral nutrition (EN) significantly reduces infectious complications, organ failure, and mortality rates [31]. Total enteral nutrition (TEN) through either a nasogastric tube or nasojejunal tube significantly reduces the rate of infections, mortality, and multi-organ failure [32]. Both the nasogastric tube and the nasojejunal tube were equally safe and effective [33]. TEN reduces pancreatic infection by maintaining the gut mucosal barrier integrity, which in turn reduces bacterial translocation from the gut. In the absence of severe nausea and vomiting, gastric outlet obstruction, ileus, or colonic pseudo-obstruction, oral feeding should be hastened as soon as the patient tolerates it. Only when the patient does not tolerate oral nutrition is EN (through NG or NJ tube) the next line of nutritional therapy. If oral and enteral nutrition are not feasible or intolerable, total parenteral nutrition (TPN) should be considered along with small amounts of enteral feeding to prevent infection.

Therapeutic interventions

**Figure 3:**
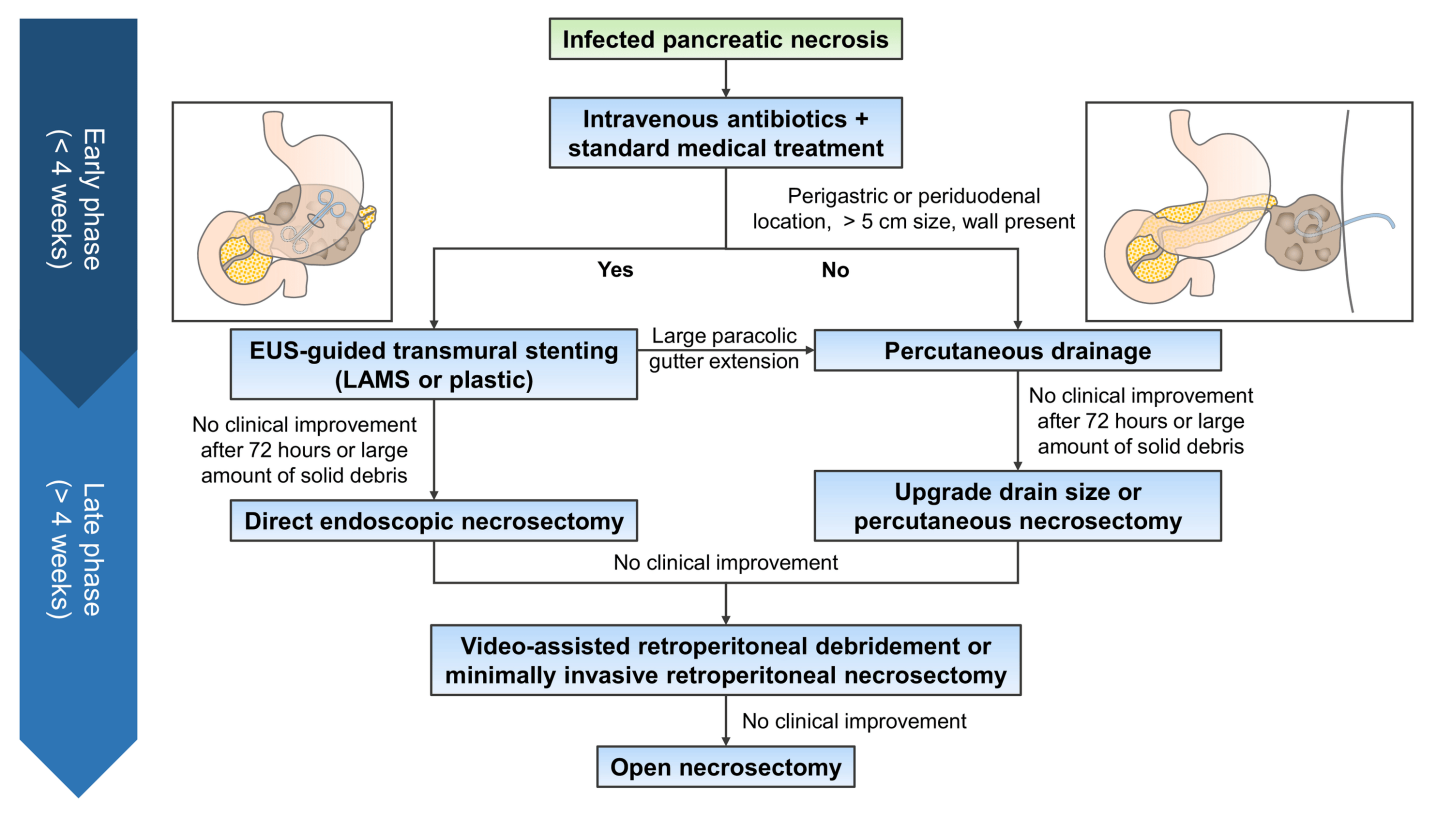
Step-wise approach to the management of infected pancreatic necrosis. EUS: endoscopic ultrasound; LAMS: lumen-apposing metal stent Image credits: Dr. Suprabhat Giri

IPN almost always requires a therapeutic drainage/debridement [13,14,34]. Therapeutic interventions for IPN are categorized into two categories: minimally invasive interventions and open surgical interventions. Compared to open surgical interventions, minimally invasive interventions are associated with significantly lower morbidity and mortality [35]. These minimally invasive procedures (MIPs) include percutaneous, endoscopic, and minimally invasive surgery (MIS), often alone or in combination. Table 1 summarizes the therapeutic modalities for the management of IPN. The current standard of care for an IPN is a "step-up" approach. A ‘step-up’ approach begins with the least invasive procedure (usually drainage) and subsequently upgrades to more invasive procedures (debridement) if indicated (Figure 3).

**Table 1:** Therapeutic modalities for the management of infected pancreatic necrosis. ANC: acute necrotic collection; WON: walled-off necrosis; ETD: endoscopic transmural drainage

Type of Intervention	Indications	Contraindications	Complications
Percutaneous Catheter Drainage (PCD)	Failed conservative treatment, with infected ANC, or infected WON (if ETD is not feasible, or deep extensions of WON)	Multiple small collections, early necrosis with non-liquefied debris, unfavorable anatomical location (e.g., interloop), uncorrected coagulopathy (INR >1.5 or platelet count <50,000/μL)	Fistula formation, bleeding, incomplete drainage, superinfection
Endoscopic Transmural Drainage (With or Without necrosectomy)	Infected peri-gastric or duodenal collections WON (>4 weeks)	Non-encapsulated collections, collections far from the GI lumen, and uncorrected coagulopathy	Bleeding, perforation, stent migration, secondary infection
Direct Endoscopic Necrosectomy (DEN)	Persistent sepsis and/or organ failure after 72 hours of ETD or large necrosis burden	Predominantly solid necrosis, multiple or multiloculated collections not communicating, presence of pseudoaneurysm or vascular involvement (↑ bleeding risk)	Bleeding, perforation, secondary infection or worsening sepsis, air embolism, stent occlusion or migration
Video-Assisted Retroperitoneal Debridement (VARD)	Failure of PCD, posteriorly located necrosis, suitable retroperitoneal access	No safe percutaneous access, unstable patient	Bleeding, fistulae, incisional hernia, incomplete necrosectomy
Open Surgical Necrosectomy	Last resort, severe clinical deterioration, and failure of all other methods	Critically ill patients unfit for surgery	High morbidity and mortality, multi-organ failure

Percutaneous Drainage (PCD)

When intervention is indicated in the early acute period, as in cases of persistent organ failure and features of sepsis, PCD is the preferred initial therapeutic intervention, as a wall might not have been formed. The majority of patients with IPN benefit from PCD alone. PCD has been beneficial for both central and peripheral infected pancreatic fluid collections. Walled-off necrosis (WON) with deep extension and complex WON also utilize PCD to maximize drainage. For encapsulated peri-gastric or peri-duodenal collections with deep extension, a combination of PCD and ED is performed (combined drainage) [14].

**Figure 4:**
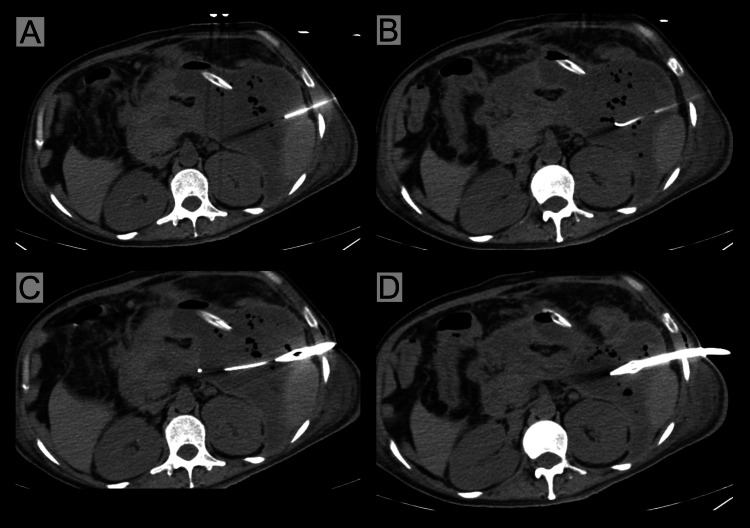
Computed tomography-guided percutaneous drainage of infected pancreatic necrosis. (A) Needle insertion, (B) guidewire insertion, (C) catheter insertion after tract dilatation, and (D) final position of the catheter Image credits: Dr. Swati Das Patients' consent was taken for the publication of the image.

PCD can be performed under ultrasound or CT guidance (Figure 4). The advantage of ultrasound-guided PCD is that it can be done at the bedside. PCD starts with a catheter size of 12-14 Fr and is usually upgraded to a size of 20 Fr. The clinical success rate of PCD in IPN varies from 18% to 89% [36]. In approximately 50% of patients with infected necrosis, PCD alone demonstrated clinical success and prevented the need for additional necrosectomy. The clinical success rate of PCD depends on the severity of the ANP and the size and distribution of the peripancreatic collections. Infected collections with significant liquefied debris showed a hasty response to PCD, which can be observed by a substantial reduction in collection size within two weeks [37]. Various factors associated with a predicted catheter-drainage failure are male sex, predicted BMI >25, BISAP score ≥ 4, a higher percentage of necrotic pancreatic tissue, heterogeneity of the necrotic collection, and multiple organ failure (specifically respiratory failure onset within 24 hours before catheter drainage) [38,39].

The POINTER trial assigned 104 patients with IPN to either immediate drainage or postponed drainage of IPN. The immediate drainage group had catheter drainage within 24 hours of randomization, and the postponed group had catheter drainage after the IPN had become largely or fully encapsulated. This postponed approach resulted in fewer interventions than immediate drainage (2.6 versus 4.4, mean difference, 1.8; 95% CI: 0.6-3.0), with the added benefit of one-third of patients with IPN improving with antibiotics only and never requiring an intervention. The rate of complications did not significantly differ between the groups [11]. The long-term follow-up data of the same trial showed that after the first six months of follow-up, both groups had similar primary outcomes (death or major complications). The postponed group continued to have the benefit of fewer additional drainage procedures (7% versus 15%, p = 0.34) and, overall, significantly fewer total interventions (4 vs. 1, p = 0.001). The pancreatic function and quality of life were no different between the groups. The POINTER trial and its long-term follow-up data were in favor of a postponed approach in IPN, given fewer interventions and clinical resolution only with antibiotics in a significant proportion of patients [40].

Vigorous irrigation of the necrotic cavity is required with 100-200 mL of normal saline or distilled water every eight hours. If there is no clinical improvement or the patient deteriorates after 72 hours of PCD, it may be necessary to perform repeat imaging, put additional percutaneous drains, or upgrade the existing drain. The number and size of catheters had no impact on PCD outcome, which may be explained by the fact that spontaneous liquefaction of solid debris in the natural course is accelerated by aggressive irrigation [41,42]. Necrosectomy is required if the patient remains symptomatic or deteriorates even after optimal percutaneous drain placement. In a study, more than half of the cases required a necrosectomy after PCD, which may be percutaneous, video-assisted retroperitoneal debridement (VARD), retroperitoneal necrosectomy, or surgical necrosectomy [43]. The higher mean computed tomographic density and distribution range of infected pancreatic necrosis are predictors of necrosectomy. However, these patients are usually very sick and should be managed by a multidisciplinary team that decides on additional interventions based on the clinical condition of the patient and the expected clinical outcome. Debridement of the infected solid debris accentuates clinical recovery in patients with IPN with partial response or nonresponse to PCD. The target of necrosectomy is the clinical resolution of symptoms along with complete debridement of necrotic tissue as well as the appearance of healthy granulation tissue.

The complications of PCD are frequent clogging of the catheters, external pancreatic fistulas (EPF), hollow viscus injury, hemorrhage, and death. One-fourth of patients treated with PCD had complications, the most common being EPF. The overall mortality rate of PCD was 18%, and the commonest cause of death was multi-organ failure [36]. A study by Gupta et al. found that hollow-viscus and vascular injuries are major complications of PCD in the step-up approach of pancreatic necrosis. PCD-induced hollow-viscus injuries were colonic fistula (~70%), duodenal, and jejunal fistulas. Most (~80%) of the fistulae improved with conservative treatment by withdrawal of the drain. Similarly, bleeding due to pseudoaneurysm was managed by conservative treatment, angioembolization (~20%), and surgery (~35%) [44].

Sinus Tract Endoscopy (STE)

STE is a minimally invasive technique for infected pancreatic or peripancreatic necrosis debridement. It is also known as percutaneous direct endoscopic necrosectomy (PDEN). STE is indicated for IPN, which does not improve after PCD and is laterally placed (> 10 mm from the gastric or duodenal wall) or has deep (paracolic gutter or pelvic) extensions of IPN. The steps of STE are initial PCD, tract dilatation and maturation, endoscopic necrosectomy (with an adult gastroscope), and post-procedure replacement of the drain [45]. Metallic stents (esophageal fully covered self-expandable metal stents) are sometimes used to keep the tract open. Multiple sessions of STE every two to five days are required to remove the necrotic tissue completely. The clinical success rate of STE ranges from 75% to 86%, with a complication rate of 25%-88% [46-48].

Endoscopic Drainage (ED) and Direct Endoscopic Necrosectomy (DEN)

**Figure 5:**
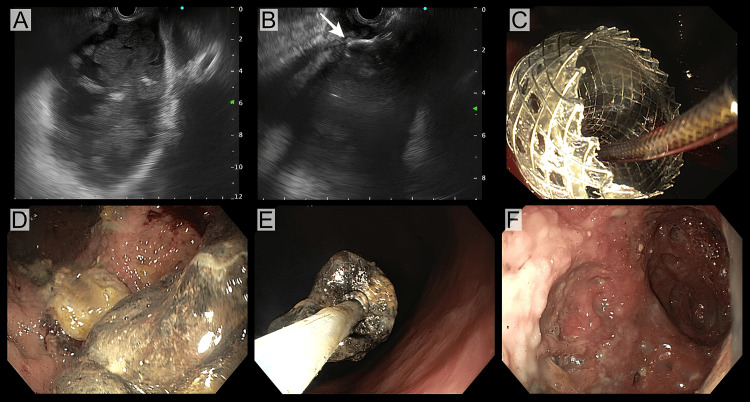
Endoscopic cystogastrostomy and necrosectomy. (A) visualized walled-off necrosis with predominantly solid debris inside, (B) puncture with electrocautery-enhanced lumen-apposing metal stent with deployement of distal flange (arrow) under ultrasound view, (C) deployment of proximal flange inside the stomach, (D) solid debris inside the cavity, (E) removal of solid debris using snare, and (F) near complete removal of solid debris after three session of necrosectomy. Image credits: Dr. Suprabhat Giri Patients' consent was taken for the publication of the image.

EUS-guided transmural drainage (EUS-TD) is the preferred ED method for peri-gastric or peri-duodenal infected WON. EUS-TD is performed in the following four steps: transmural puncture of the PFC with an FNA needle, guidewire placement, transmural tract dilatation, and stent placement. Multiple plastic stents or metallic stents are used to bridge the fistula for drainage [14,49]. Large diameter (>15 mm to 20 mm) lumen apposing metal stents (LAMS) are preferred for EUS-TD [50]. Electrocautery-enhanced LAMS (EC-LAMS) are technically easier to deploy and can be deployed with or without guidewire placement (the "free-hand" technique) (Figure 5). A retrospective study reported the factors predicting step-up necrosectomy and/or additional drains (endoscopic or percutaneous) in patients undergoing ED, and these are pancreatic fluid collections ≥ 10 cm, paracolic extension, or ≥ 30% necrosis [51].

There are multiple technical variations in EUS-TD that need to be discussed. With respect to the type of stent for IPN, a meta-analysis of randomized studies reported no difference in the technical and clinical success and the need for reintervention. However, the use of metal stents was associated with a shorter procedural duration and a higher cost [52]. Thus, either of the stents can be used for the ED of IPN. Concerning the use of coaxial plastic stent within LAMS for pancreatic fluid collection, a meta-analysis did not show any difference in the clinical success [53]. However, an RCT including only patients with WON reported a significantly lower risk of overall adverse events and stent occlusion with a coaxial plastic stent within the LAMS [54]. Thus, in the case of IPN, the coaxial stent may be preferred, but further studies are required before the recommendation.

A "step-up" DEN is recommended if, after 72 hours of drainage, there is no clinical improvement or presence of clinical deterioration (Figure 5). This technique was first reported by Seifert et al. in three patients with IPN [55]. Seewald et al. also showed successful resolution of pancreatic necrosis and abscesses in nine patients by aggressive endoscopic therapy (a combination of ED and necrosectomy) with minimal complications [56]. Data from a multicenter study showed a high success rate of DEN with persistent long-term follow-up results. Of the 93 patients who underwent DEN after acute pancreatitis, 80% had a successful clinical resolution in a month. Of these patients, 84% sustained clinical remission, and 16% developed recurrent acute pancreatitis on long-term follow-up. The one-month mortality and complication rates were 7.5% and 26%, respectively [57]. In stable patients with encapsulated IPN, upfront necrosectomy lowers intervention needs for treatment success. In upfront necrosectomy, direct necrosectomy is followed by stenting in the same session [58]. The target of necrosectomy is complete resolution of symptoms, with complete debridement of the necrotic tissue.

Adjuvant Techniques Along With EUS‑Guided Drainage of IPN

A naso-cystic catheter placed alongside plastic or through the LAMS can be used for irrigation of the necrotic cavity. One study showed that irrigation using a naso-cystic tube reduced the risk of stent blockage (33% vs. 13%, p = 0.03) [59]. However, the routine use of a naso-cystic catheter needs further prospective studies.

The use of hydrogen peroxide (H_2_O_2_) has been reported during the DEN of WON. H_2_O_2_ decomposes into oxygen and water upon contact with organic tissues, generating a soft foam that facilitates the removal of necrotic tissue. Additionally, it may enhance wound healing by inducing granulation and fibrosis. In a meta-analysis of retrospective studies, the pooled clinical success rate was 91.6%, with an overall AE rate of 19.3%, and no adverse events were directly attributed to H_2_O_2_ [60]. However, there is no standardization of this technique with significant variation in the strength (0.1%-3%) and amount of H_2_O_2_ (20 mL to 1 L) used.

Over-the-scope-grasper (OTSG) is a recently introduced dedicated device for DEN that allows for the grasping of larger pieces of tissue or necrotic debris. In a retrospective analysis of the use of OTSG in 37 cases of DEN, technical success was achieved in 97% of the cases [61]. However, a prospective comparative study with standard necrosectomy tools is still pending.

The powered endoscopic debridement (PED) system contains a motorized catheter, which simultaneously suctions, cuts, and debrides tissue using negative pressure. In a prospective, multicentric study of 30 patients undergoing DEN using PED, no device-related adverse events were reported. Around half of the patients required a single session, and two-thirds required fewer than two sessions for complete debridement [62]. Thus, the PED system has the potential to reduce the number of reinterventions and duration of hospitalization, but requires future studies.

Minimally Invasive Surgery

VARD is a minimally invasive surgical technique performed in patients with IPN. For patients with IPN who do not improve after optimal PCD, VARD is indicated ("step-up" treatment). The left retroperitoneum is the most preferred route for PCD, and the drain acts as a guide for the VARD procedure. In VARD, only the loose necrotic tissue is removed under direct vision or using a videoscope. It decreases postoperative morbidity and mortality and avoids major laparotomies. The clinical success of VARD ranges from 60% to 93%, with a high risk of complications (24%-54%) [63].

Step-up approaches

Infected necrotizing pancreatitis entails a high risk of mortality and many complications. The "step-up" strategy involves initial drainage (percutaneous/endoscopic) followed by, if necessary, minimally invasive necrosectomy (surgical/endoscopic).

Open Necrosectomy Versus Step-Up Approach

The PANTER trial challenged the concept of open necrosectomy in IPN and introduced the minimally invasive "step-up" approach. In this trial, 88 patients with necrotizing pancreatitis were randomly assigned to primary open necrosectomy or a step-up approach. The step-up approach included drainage and retroperitoneal necrosectomy if needed. In the step-up group, one-third of patients never required a necrosectomy. Major complications, organ failures, diabetes, and incisional hernias were lower in the step-up group than in the open necrosectomy group, with similar death rates [2]. The long-term follow-up (mean: 86 months ± 11 months) data of the patients in the PANTER trial also showed that the step-up group experienced significantly fewer deaths (44% versus 73%, p = 0.005), incisional hernias, pancreatic exocrine, or endocrine insufficiency. However, no significant difference was observed between the groups in terms of the requirement of additional drainage procedures (11% vs. 13%, p = 0.99), or pancreatic surgery (11% vs. 5%, p = 0.43), or in the incidence of recurrent acute pancreatitis, or chronic pancreatitis [64]. Thus, the results of the PANTER trial and its long-term follow-up data advocate the step-up approach for IPN. 

Endoscopic Step-Up Versus Surgical Step-Up Approach

The TENSION trial favored the endoscopic step-up approach over the surgical step-up approach. The endoscopic approach included EUS-guided drainage and endoscopic necrosectomy as needed. The surgical method involved PCD initially and VARD in non-responders. The study did not find a significant difference in the rate of major complications or mortality within six months but noted fewer pancreatic fistulas in the endoscopic group [65]. The long-term follow-up data of the TENSION trial (ExTENSION study) patients also revealed that, past the initial six-month follow-up period, the endoscopy group had lower rates of pancreaticocutaneous fistulas (8% vs. 34%) and required fewer interventions (7% vs. 24%) compared to the surgery group, with no significant differences in death or major complications observed between the groups [66]. The TENSION trial and its long-term follow-up data showed that endoscopic step-ups have an edge over surgical step-ups, given the low rate of pancreaticocutaneous fistulas. A systematic review and network meta-analysis also ranked the safety of various invasive treatments for an IPN based on the surface under the cumulative ranking (SUCRA). The endoscopic step-up approach with endoscopic debridement (SUCRA 87.1%) was the safest, followed by the surgical step-up (SUCRA 59.5%). It disfavored the other invasive approaches like early surgical debridement, peritoneal lavage, and delayed surgical debridement [67].

MISER trial compared MIS (VARD or laparoscopic) with endoscopic step-up approach depending on the location of the collection in patients with IPN. Endoscopic step-up approach was found to be superior to minimal invasive surgery in terms of a lower incidence of enteral or pancreatocutaneous fistulae (28% versus 0%, p = 0.001), a lower mean number of major complications, and a better quality of life at a lower cost [68]. The PENGUIN trial showed that significantly better clinical outcomes were observed with endoscopic necrosectomy than with surgical necrosectomy. Twenty patients who underwent either of the interventions, in which surgical necrosectomy included VARD or laparotomy if needed. Endoscopic necrosectomy showed significantly lower post-procedural IL-6 levels vs. surgery (p = 0.004) and decreased occurrence of the composite clinical endpoint of major complications (20% vs. 80%, p = 0.03). The incidence of pancreatic fistulas was significantly lower (10% vs. 70%, p = 0.02), with no new-onset post-procedure organ failure (0% vs. 50%, p = 0.03) in the endoscopic group [69]. A systematic review and meta-analysis published in 2020 reviewed three RCTs among 190 patients with IPN and found that, compared to the surgical group, the endoscopic group experienced lower odds of new organ failure, organ perforations or fistulae, and shorter hospital stays with a similar mortality rate. The study also reported that no significant differences were found in bleeding that needed intervention, incisional hernia, and exocrine or endocrine insufficiency [70]. Table 2 summarizes the advantages and disadvantages of the endoscopic and surgical step-up approach.

**Table 2:** Advantages and disadvantages of endoscopic and surgical step-up approaches.

	Endoscopic step-up approach	Surgical step-up approach
Advantages	No need of abdominal wall incision and associated adverse events (e.g., pancreticocutaneous fistula, incisional hernia, wound infection, etc.)	Necrotic collection is percutaneously accessible in a majority
	Reduced pro-inflammatory response as measured by lower post-procedural interleukin-6 levels	High effectiveness in removing large pieces of necrotic tissue
	Shorter hospital stay	
	Fewer reinterventions on long-term follow-up	
Disadvantages	Peripherally located collections and those without complete walls are not accessible	Risk of pancreatocutaneous fistula
	Multiple procedures are needed for a complete necrosectomy	Requires general anesthesia
	Availability of expertise	Longer hospital stay

Knowledge Gaps and Future Perspectives

There are no reliable clinical or laboratory parameters to diagnose or predict the development of IPN with high accuracy. While procalcitonin shows promise, its clinical value is still debated, and its heterogeneity across studies is noted. Gas on CT scan, a specific sign, has low sensitivity. FNA has high false-negative rates, limiting its usefulness in all suspected cases. Larger multi-centric studies are required to validate the clinical use of PET/CT with 18F-FDG-labeled autologous leukocytes for the diagnosis of IPN [71]. The added value of diffuse-weighted MRI in detecting infected pancreatic fluid collections needs further demonstration [72]. Future prospective or randomized studies should focus on the optimal timing of PCD or EUS-TD in patients with IPN, comparing immediate versus delayed drainage until a wall is established. There is also a need for further studies to determine the choice of stent (plastic vs. metal) for EUS-TD, the need for a coaxial stent inside LAMS, and the role of adjuvant techniques such as OTSG and PED during DEN.

## Conclusions

IPN is a common occurrence in patients with ANP and can develop either late (common) or early in the course. The diagnosis of infected necrosis is based upon clinical features, positive Gram stain or culture, and/or the presence of bubble sign on an abdominal CT scan. Clinical indicators include fever, persistent unwellness, clinical deterioration, bacteremia, or worsening laboratory markers of inflammation. The primary management of IPN is source control through drainage and/or debridement, with initiation of antibiotics once IPN is strongly suspected or confirmed. Therapeutic interventions for IPN are categorized into minimally invasive and open surgical interventions. The current standard of care for an IPN is a step-up approach, starting with the least invasive procedure (usually drainage) and subsequently upgrading to more invasive procedures (debridement) if necessary. PCD or EUS-TD are the preferred initial therapeutic interventions for IPN in cases of persistent organ failure and features of sepsis. Necrosectomy is required if the patient remains symptomatic or deteriorates even after optimal percutaneous drain placement. Surgical step-up should be considered if endoscopic therapy fails to achieve adequate drainage or debridement or if there are endoscopically inaccessible collections. IPN is a life-threatening disease, and outcomes can be optimized by a multidisciplinary approach.

## References

[1] Kokosis G, Perez A, Pappas TN (2014). Surgical management of necrotizing pancreatitis: an overview. World J Gastroenterol.

[2] van Santvoort HC, Besselink MG, Bakker OJ (2010). A step-up approach or open necrosectomy for necrotizing pancreatitis. N Engl J Med.

[3] Petrov MS, Shanbhag S, Chakraborty M, Phillips AR, Windsor JA (2010). Organ failure and infection of pancreatic necrosis as determinants of mortality in patients with acute pancreatitis. Gastroenterology.

[4] van Dijk SM, Hallensleben ND, van Santvoort HC, Fockens P, van Goor H, Bruno MJ, Besselink MG (2017). Acute pancreatitis: recent advances through randomised trials. Gut.

[5] Reuken PA, Albig H, Rödel J, Hocke M, Will U, Stallmach A, Bruns T (2018). Fungal infections in patients with infected pancreatic necrosis and pseudocysts: risk factors and outcome. Pancreas.

[6] Singh RR, Mitchell W, David Y (2021). Pancreatic fungal infection in patients with necrotizing pancreatitis: a systematic review and meta-analysis. J Clin Gastroenterol.

[7] Tenner S, Vege SS, Sheth SG (2024). American College of Gastroenterology guidelines: management of acute pancreatitis. Am J Gastroenterol.

[8] Petrov MS, Chong V, Windsor JA (2011). Infected pancreatic necrosis: not necessarily a late event in acute pancreatitis. World J Gastroenterol.

[9] Tan C, Yang L, Shi F (2020). Early systemic inflammatory response syndrome duration predicts infected pancreatic necrosis. J Gastrointest Surg.

[10] Padhan RK, Jain S, Agarwal S (2018). Primary and secondary organ failures cause mortality differentially in acute pancreatitis and should be distinguished. Pancreas.

[11] Boxhoorn L, van Dijk SM, van Grinsven J (2021). Immediate versus postponed intervention for infected necrotizing pancreatitis. N Engl J Med.

[12] van Baal MC, Bollen TL, Bakker OJ (2014). The role of routine fine-needle aspiration in the diagnosis of infected necrotizing pancreatitis. Surgery.

[13] Leppäniemi A, Tolonen M, Tarasconi A (2019). 2019 WSES guidelines for the management of severe acute pancreatitis. World J Emerg Surg.

[14] Baron TH, DiMaio CJ, Wang AY, Morgan KA (2020). American Gastroenterological Association clinical practice update: management of pancreatic necrosis. Gastroenterology.

[15] Giri S, Das S, Nemani P, Mohanty SK, Nath P, Mohapatra V (2024). Does the site, size, and number of necrotic collections affect the outcome of necrotizing pancreatitis? - a prospective analysis. Emerg Radiol.

[16] Bugiantella W, Rondelli F, Boni M, Stella P, Polistena A, Sanguinetti A, Avenia N (2016). Necrotizing pancreatitis: a review of the interventions. Int J Surg.

[17] Mofidi R, Suttie SA, Patil PV, Ogston S, Parks RW (2009). The value of procalcitonin at predicting the severity of acute pancreatitis and development of infected pancreatic necrosis: systematic review. Surgery.

[18] Alberti P, Pando E, Mata R (2022). The role of procalcitonin as a prognostic factor for acute cholangitis and infections in acute pancreatitis: a prospective cohort study from a European single center. HPB (Oxford).

[19] Samanta J, Dhar J, Birda CL (2023). Dynamics of serum procalcitonin can predict outcome in patients of infected pancreatic necrosis: a prospective analysis. Dig Dis Sci.

[20] Tarján D, Szalai E, Lipp M (2024). Persistently high procalcitonin and C-reactive protein are good predictors of infection in acute necrotizing pancreatitis: a systematic review and meta-analysis. Int J Mol Sci.

[21] Siriwardena AK, Jegatheeswaran S, Mason JM (2022). A procalcitonin-based algorithm to guide antibiotic use in patients with acute pancreatitis (PROCAP): a single-centre, patient-blinded, randomised controlled trial. Lancet Gastroenterol Hepatol.

[22] Fritz S, Hackert T, Hartwig W (2010). Bacterial translocation and infected pancreatic necrosis in acute necrotizing pancreatitis derives from small bowel rather than from colon. Am J Surg.

[23] Ning C, Huang G, Shen D (2019). Adverse clinical outcomes associated with multidrug-resistant organisms in patients with infected pancreatic necrosis. Pancreatology.

[24] Mowbray NG, Ben-Ismaeil B, Hammoda M, Shingler G, Al-Sarireh B (2018). The microbiology of infected pancreatic necrosis. Hepatobiliary Pancreat Dis Int.

[25] Garg PK, Madan K, Pande GK, Khanna S, Sathyanarayan G, Bohidar NP, Tandon RK (2005). Association of extent and infection of pancreatic necrosis with organ failure and death in acute necrotizing pancreatitis. Clin Gastroenterol Hepatol.

[26] Loganathan PK, Muktesh G, Kochhar R, Samanta J, Shah J, Angrup A (2024). Natural history and microbiological profiles of patients with acute pancreatitis with suspected infected pancreatic necrosis. Cureus.

[27] Lu JD, Cao F, Ding YX, Wu YD, Guo YL, Li F (2019). Timing, distribution, and microbiology of infectious complications after necrotizing pancreatitis. World J Gastroenterol.

[28] Pezzilli R, Zerbi A, Campra D (2015). Consensus guidelines on severe acute pancreatitis. Dig Liver Dis.

[29] Wolbrink DR, Kolwijck E, Ten Oever J, Horvath KD, Bouwense SA, Schouten JA (2020). Management of infected pancreatic necrosis in the intensive care unit: a narrative review. Clin Microbiol Infect.

[30] Reboli AC, Rotstein C, Pappas PG (2007). Anidulafungin versus fluconazole for invasive candidiasis. N Engl J Med.

[31] Ramanathan M, Aadam AA (2019). Nutrition management in acute pancreatitis. Nutr Clin Pract.

[32] Liu M, Gao C (2021). A systematic review and meta-analysis of the effect of total parenteral nutrition and enteral nutrition on the prognosis of patients with acute pancreatitis. Ann Palliat Med.

[33] Kumar A, Singh N, Prakash S, Saraya A, Joshi YK (2006). Early enteral nutrition in severe acute pancreatitis: a prospective randomized controlled trial comparing nasojejunal and nasogastric routes. J Clin Gastroenterol.

[34] Sahu SK, Mallick B, Nath P, Praharaj D, Giri S, Panigrahi SC, Anand AC (2025). Management of pancreatic fluid collections: a concise review. J Integrat Med Res.

[35] Wroński M, Cebulski W, Słodkowski M, Krasnodębski IW (2014). Minimally invasive treatment of infected pancreatic necrosis. Prz Gastroenterol.

[36] Ke L, Li J, Hu P, Wang L, Chen H, Zhu Y (2016). Percutaneous catheter drainage in infected pancreatitis necrosis: a systematic review. Indian J Surg.

[37] Hollemans RA, Bollen TL, van Brunschot S (2016). Predicting success of catheter drainage in infected necrotizing pancreatitis. Ann Surg.

[38] Sundaram Venkatesan G, Thulasiraman S, Kesavan B, Saravanan D, Chinnaraju N (2022). Predicting the success of catheter drainage in infected necrotising pancreatitis: a cross-sectional observational study. Cureus.

[39] Garret C, Douillard M, David A (2022). Infected pancreatic necrosis complicating severe acute pancreatitis in critically ill patients: predicting catheter drainage failure and need for necrosectomy. Ann Intensive Care.

[40] Van Veldhuisen CL, Sissingh NJ, Boxhoorn L (2024). Long-term outcome of immediate versus postponed intervention in patients with infected necrotizing pancreatitis (POINTER): multicenter randomized trial. Ann Surg.

[41] Tong Z, Li W, Yu W (2012). Percutaneous catheter drainage for infective pancreatic necrosis: is it always the first choice for all patients?. Pancreas.

[42] Bruennler T, Langgartner J, Lang S (2008). Outcome of patients with acute, necrotizing pancreatitis requiring drainage-does drainage size matter?. World J Gastroenterol.

[43] Cao X, Cao F, Li A (2017). Predictive factors of pancreatic necrosectomy following percutaneous catheter drainage as a primary treatment of patients with infected necrotizing pancreatitis. Exp Ther Med.

[44] Gupta R, Kulkarni A, Babu R (2020). Complications of percutaneous drainage in step-up approach for management of pancreatic necrosis: experience of 10 years from a tertiary care center. J Gastrointest Surg.

[45] Poves I, Burdío F, Dorcaratto Dorcaratto, Dimitri D, Grande L (2014). Minimally invasive techniques in the treatment of severe acute pancreatitis. Open Med.

[46] Carter CR, McKay CJ, Imrie CW (2000). Percutaneous necrosectomy and sinus tract endoscopy in the management of infected pancreatic necrosis: an initial experience. Ann Surg.

[47] Connor S, Ghaneh P, Raraty M (2003). Minimally invasive retroperitoneal pancreatic necrosectomy. Dig Surg.

[48] Raraty MG, Halloran CM, Dodd S (2010). Minimal access retroperitoneal pancreatic necrosectomy: improvement in morbidity and mortality with a less invasive approach. Ann Surg.

[49] Giri S, Bhrugumalla S, Gangadhar S, Angadi S (2024). Comparative outcome of single versus two double-pigtail stents for endoscopic drainage of pancreatic fluid collections with minimal necrosis: a retrospective analysis. Acta Gastroenterol Belg.

[50] Sundaram S, Giri S, Binmoeller K (2024). Lumen-apposing metal stents: a primer on indications and technical tips. Indian J Gastroenterol.

[51] Chandrasekhara V, Elhanafi S, Storm AC (2021). Predicting the need for step-up therapy after EUS-guided drainage of pancreatic fluid collections with lumen-apposing metal stents. Clin Gastroenterol Hepatol.

[52] Zafar Y, Sohail MU, Ibrahim ZS (2025). Efficacy of metal stents versus plastic stents for treatment of walled-off pancreatic necrosis: a systematic review and meta-analysis. JGH Open.

[53] Giri S, Harindranath S, Afzalpurkar S, Angadi S, Sundaram S (2023). Does a coaxial double pigtail stent reduce adverse events after lumen apposing metal stent placement for pancreatic fluid collections? A systematic review and meta-analysis. Ther Adv Gastrointest Endosc.

[54] Vanek P, Falt P, Vitek P (2023). EUS-guided transluminal drainage using lumen-apposing metal stents with or without coaxial plastic stents for treatment of walled-off necrotizing pancreatitis: a prospective bicentric randomized controlled trial. Gastrointest Endosc.

[55] Seifert H, Wehrmann T, Schmitt T, Zeuzem S, Caspary WF (2000). Retroperitoneal endoscopic debridement for infected peripancreatic necrosis. Lancet.

[56] Seewald S, Groth S, Omar S (2005). Aggressive endoscopic therapy for pancreatic necrosis and pancreatic abscess: a new safe and effective treatment algorithm (videos). Gastrointest Endosc.

[57] Seifert H, Biermer M, Schmitt W (2009). Transluminal endoscopic necrosectomy after acute pancreatitis: a multicentre study with long-term follow-up (the GEPARD study). Gut.

[58] Bang JY, Lakhtakia S, Thakkar S (2024). Upfront endoscopic necrosectomy or step-up endoscopic approach for infected necrotising pancreatitis (DESTIN): a single-blinded, multicentre, randomised trial. Lancet Gastroenterol Hepatol.

[59] Siddiqui AA, Dewitt JM, Strongin A (2013). Outcomes of EUS-guided drainage of debris-containing pancreatic pseudocysts by using combined endoprosthesis and a nasocystic drain. Gastrointest Endosc.

[60] Mohan BP, Madhu D, Toy G (2022). Hydrogen peroxide-assisted endoscopic necrosectomy of pancreatic walled-off necrosis: a systematic review and meta-analysis. Gastrointest Endosc.

[61] Brand M, Bachmann J, Schlag C (2022). Over-the-scope-grasper: a new tool for pancreatic necrosectomy and beyond - first multicenter experience. World J Gastrointest Surg.

[62] Stassen PM, de Jonge PJ, Bruno MJ (2022). Safety and efficacy of a novel resection system for direct endoscopic necrosectomy of walled-off pancreas necrosis: a prospective, international, multicenter trial. Gastrointest Endosc.

[63] Budkule D, Desai G, Pande P, Narkhede R, Wagle P, Varty P (2019). An outcome analysis of videoscopic assisted retroperitoneal debridement in infected pancreatic necrosis: a single centre experience. Turk J Surg.

[64] Hollemans RA, Bakker OJ, Boermeester MA (2019). Superiority of step-up approach vs open necrosectomy in long-term follow-up of patients with necrotizing pancreatitis. Gastroenterology.

[65] van Brunschot S, van Grinsven J, van Santvoort HC (2018). Endoscopic or surgical step-up approach for infected necrotising pancreatitis: a multicentre randomised trial. Lancet.

[66] Onnekink AM, Boxhoorn L, Timmerhuis HC (2022). Endoscopic versus surgical step-up approach for infected necrotizing pancreatitis (ExTENSION): long-term follow-up of a randomized trial. Gastroenterology.

[67] Ricci C, Pagano N, Ingaldi C (2021). Treatment for infected pancreatic necrosis should be delayed, possibly avoiding an open surgical approach: a systematic review and network meta-analysis. Ann Surg.

[68] Bang JY, Arnoletti JP, Holt BA (2019). An endoscopic transluminal approach, compared with minimally invasive surgery, reduces complications and costs for patients with necrotizing pancreatitis. Gastroenterology.

[69] Bakker OJ, van Santvoort HC, van Brunschot S (2012). Endoscopic transgastric vs surgical necrosectomy for infected necrotizing pancreatitis: a randomized trial. JAMA.

[70] Haney CM, Kowalewski KF, Schmidt MW (2020). Endoscopic versus surgical treatment for infected necrotizing pancreatitis: a systematic review and meta-analysis of randomized controlled trials. Surg Endosc.

[71] Bhattacharya A, Kochhar R, Sharma S, Ray P, Kalra N, Khandelwal N, Mittal BR (2014). PET/CT with 18F-FDG-labeled autologous leukocytes for the diagnosis of infected fluid collections in acute pancreatitis. J Nucl Med.

[72] Borens B, Arvanitakis M, Absil J (2017). Added value of diffusion-weighted magnetic resonance imaging for the detection of pancreatic fluid collection infection. Eur Radiol.

